# ﻿Taxonomic study on the genus *Xenicotela* Bates from China (Cerambycidae, Lamiinae, Lamiini)

**DOI:** 10.3897/zookeys.1122.86344

**Published:** 2022-09-28

**Authors:** Guanglin Xie, Maxwell V. L. Barclay, Bin Chen

**Affiliations:** 1 Institute of Entomology, College of Agriculture, Yangtze University, Jingzhou, Hubei, 434025, China Yangtze University Jingzhou China; 2 Department of Life Sciences, Natural History Museum, London, SW7 5BD, UK Natural History Museum London United Kingdom; 3 Chongqing Key Laboratory of Vector Insects, Institute of Entomology and Molecular Biology, Chongqing Normal University, Chongqing, 401331, China Chongqing Normal University Chongqing China

**Keywords:** Longhorned beetles, new combination, new species, taxonomy

## Abstract

A taxonomic review of the Chinese species of the genus *Xenicotela* Bates, 1884 is presented. A new species, *Xenicotelagriseomaculata***sp. nov.**, is described from Chongqing, China, and a new combination, *Xenicotelaconvexicollis* (Gressitt, 1942) **comb. nov.**, is proposed.

## ﻿Introduction

The genus *Xenicotela* was established based on *Xenicotelafuscula* Bates from Higo (Japan), (presently considered a synonym of *Xenicotelapardalina* (Bates, 1884)), as a result of a comparison with the similar genus *Xenolea* Thomson (Bates 1884). Up to now, three species: *X.pardalina* (Bates, 1884), *X.distincta* (Gahan, 1888), and *X.bimaculata* (Pic, 1925) are known from Japan, South Korea, China, Vietnam, Laos, Nepal, and India. Among them, only one species, *X.distincta*, has been recorded in China (Cho et al. 1963; Hubweber et al. 2010; Kariyanna et al. 2017; Lin and Tavakilian 2019; Tavakilian and Chevillotte 2021).

In the present study, a new species, *Xenicotelagriseomaculata* sp. nov., is described and illustrated from Chongqing, China. *Monochamusconvexicollis* Gressitt, 1942 is transferred to *Xenicotela* based on the examination of the holotype and three specimens from the type locality and its adjacent area.

## ﻿Materials and methods

Specimens from the following institutional collections were examined and/or photographed in this study:

**IZAS**Institute of Zoology, Chinese Academy of Sciences, Beijing, China;

**NHMUK**Natural History Museum, London, UK;

**MNHN**Muséum National d’Histoire Naturelle, Paris, France;

**SWU** Southwest University, Chongqing, China;

**CQNU**Chongqing Normal University, Chongqing, China;

**YZU** Yangtze University, Jingzhou, China;

**GZNULS** School of Life Sciences, Guizhou Normal University, Guiyang, China.

The genitalia were prepared by soaking the whole beetle in boiling water for several minutes, then opening the abdomen from the apex along the dorsopleural margin. The genitalia were then removed with fine forceps and ophthalmic scissors, and later cleared in 10% KOH at 80–100 °C for several minutes.

All habitus photographs were taken with a Canon 5D Mark II digital camera equipped with a Canon EF 100mm f/2.8L IS USM lens, and genitalia images were taken with a Leica DFC450 digital camera mounted on a Leica M205A microscope. Images of genitalia were taken by keeping them in glycerin. All images were edited using Adobe Photoshop 2020.

## ﻿Taxonomy

### ﻿Genus Xenicotela

Taxon classificationAnimaliaColeopteraCerambycidae

Bates, 1884

991F1A5E-FD09-546B-B3A2-16032F142471

Xenicotela Bates, 1884: 242; Matsushita 1933: 346; Breuning 1944: 372; Gressitt 1951: 381; Breuning 1961: 353; Rondon and Breuning 1970: 458; Makihara 2007; Hubweber et al. 2010: 288; Lin and Tavakilian 2019: 324.

#### Type species.

*Xenicotelafuscula* Bates, 1884 (= *Xenicotelapardalis* (Bates, 1884))

#### Redescription.

Body small, elongated. Eyes coarsely faceted. Antennae slender, more than 2.0 times as long as body in male and nearly 2.0 times in female; several basal antennomeres sparsely fringed ventrally, antennomeres III–XI annulated with greyish white to greyish yellow pubescence basally and apically; antennal tubercle moderately elevated; scape short, rather robust, with a narrow and completely closed cicatrix at apex, distinctly constricted near the apex; antennomere III distinctly longer than fourth, about 2.0 times as long as scape. Pronotum broader than long, anterior and posterior margin with vague transverse grooves, each side with a coniform spine at middle. Elytra elongated, with subparallel sides, apices rounded. Prosternal process lower than procoxae, arched, procoxal cavities closed posteriorly. Mesosternal process obliquely sloping anteriorly, not tuberculate, mesocoxal cavities open at side. Metasternum normal in length. Legs moderately long, femora clavate, mesotibia without groove near external apex, claw widely divergent.

#### Distribution.

Japan, South Korea, China, Vietnam, Laos, Nepal, India.

#### Comments.

The genus is characterized by the following combination of characters that distinguishes it from similar genera: antennae with basal several antennomeres (usually five segments) sparsely fringed with short setae ventrally, antennomeres III–XI annulated with greyish white to greyish yellow pubescence basally and apically; scape with a narrow and completely closed cicatrix at apex and distinctly constricted before it; lateral spine of pronotum coniform, short; mesosternal process obliquely sloping anteriorly, not tuberculate; mesotibia without groove near external apex.

Aurivillius (1922) placed the genus *Xenicotela* in the tribe Dorcaschematini. Subsequently, Matsushita (1933) defined the tribe Xenicotelini for the genus according to the following differences on the basis of comparing with tribes Ancylonotini and Prosopocerini: scape with a completely closed apical cicatrix and mesotibia without a groove near the external apex. Breuning (1943) transferred the genus into Agninii, and Gressitt (1951) placed it in Lamiini. Kariyanna et al. (2017) and Tavakilian and Chevillotte (2021) followed Matsushita’s decision and put the genus into Xenicotelini in their Cerambycidae database. In the present study, we follow Breuning’s and Gressitt’s arrangement, since the characters of *Xenicotela* correspond well with Lamiini.

### ﻿ Xenicotela distincta

Taxon classificationAnimaliaColeopteraCerambycidae

(Gahan, 1888)

417F2FF3-5B66-5220-B4D0-2E1FC10CD6C1

Figs 1 , 2 , 7 , 8 , 10 , 12 , 13 , 22 , 23

Monohammus distinctus Gahan, 1888: 392; Aurivillius 1922: 95. Type locality: Assam, India.
Xenicotela distincta : Breuning 1944: 373; Gressitt 1951: 382; Rondon and Breuning 1970: 458; Hubweber et al. 2010: 288; Weigel et al. 2013: 288; Kariyanna et al. 2017: 253; Lin and Tavakilian 2019: 324.
Nephelotus 4-maculatus Pic, 1925: 16. Type locality: Tonkin, Vietnam.
Nephelotus tonkineus Pic, 1926: 143. Type locality: Hoa-Binh, Vietnam.
Xenicotela distincta m. *tonkinensis* Breuning, 1944: 373.
Monochamus binigricollis : Wang 1998: 599, misidentification.

#### Type material examined.

***Holotype*** of *Monohammusdistinctus* Gahan (NHMUK), the label details are shown in Fig. 7. ***Holotype*** of *Nephelotustonkineus* Pic (MNHN), the label details are shown in Fig. 10.

**Figures 1, 2.**
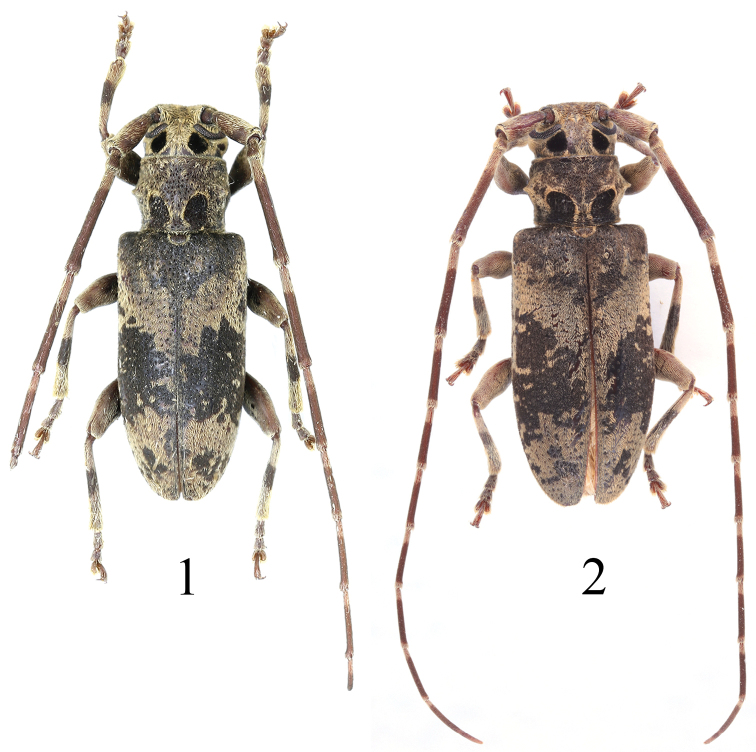
Habitus of *Xenicoteladistincta* (Gahan, 1888) **1** male **2** female **1** from Yunnan: Jiangcheng **2** from Guizhou: Ziyun.

#### Other materials examined.

One male, China: Yunnan Province, Cangyuan County, Daheishan, alt. 2400 m, May 15, 1980, coll. by Kaiquan Li (SWU); One female, China: Guizhou Province, Ziyun County, Nazuo Village, June 8, 2019, coll. by Shulin Yang (GZNULS); one female, China: Yunnan Province, Xishuangbanna Prefecture, Danuoyou, May 29, 2008, coll. by Meiying Lin (IZAS); one male, China: Yunnan Province, Jiangcheng County, Qushui Township, alt. 564 m, 22°37'1"N, 102°9'49"E, June 8, 2019, coll. by Lanbin Xiang (YZU).

#### Redescription.

**Male.** Body length 10.0 mm, humeral width 3.4 mm. Body mostly black brown to black, densely clothed with greyish yellow and black pubescence forming markings. Antennae dull reddish brown, scape and extreme apex of pedicel clothed with greyish yellow pubescence, base and extreme apex of antennomeres III–XI annulated with greyish yellow pubescence. Head provided with two slightly quadrate black pubescent spots behind upper lobes of eyes, pronotum also provided with two suboval black spots of the same texture at the posterior half, distinctly edged with greyish yellow pubescence and widely separated anteriorly. Scutellum bordered by greyish yellow pubescence. Elytra mostly black brown to black at base, with a broad transverse black band intermingled with some irregular small greyish yellow pubescent spots at middle, mostly clothed with greyish yellow pubescence intermingled with some irregular black spots at apex. Ventrites I–IV fringed with long setae at posterior edge. Legs with femora and tibiae black brown medially, with a greyish yellow pubescent ring at base and apex.

Head finely and densely punctate, frons transverse, lower lobe of eyes about as long as gena. Pronotum broader than long, deeply and slightly densely punctate, lateral spine short and small. Scutellum short, ligulate. Elytra elongate, about 2.1 times as long as width across humeri, subparallel in basal two-thirds, gradually narrowed backwards in apical third, apices slightly transversely truncate, surface deeply and slightly coarsely punctate. Legs relatively short, claws divaricate.

**Female.** Similar to male, body sometimes mostly reddish brown; antennae relatively short; lateral spine of pronotum larger than that of male; elytra about 2.0 times as long as humeral width, median band sometimes interrupted by a sutural pubescent strip.

***Male genitalia*.** Tergite VIII (Figs 12, 13) with both sides relatively circularly converge to apex, apex slightly truncated, clothed with short to medium straight setae along apical and lateral sides. Tegmen (Figs 22, 23) length approximately 1.73 mm, maximum width of ringed part approximately 0.66 mm, each paramere length approximately 0.37 mm, basal width approximately 0.19 mm; parameres widely separated at apex, with length-width ratio of each lobe about 1.95, rounded at apex, about apical two-fifths clothed with sparse setae of different lengths and thicknesses. Median lobe (Figs 22, 23) slightly longer than tegmen, obviously arcuate in lateral view, apical margin of dorsal plate and ventral plate nearly straight; median struts relatively broad, about two-fifths length of median lobe.

#### Distribution.

China (Yunnan, Guizhou), India, Vietnam, Nepal, Laos.

#### Comments.

Wang (1998) first recorded *Monochamusbinigricollis* Breuning, 1965 from China based on specimens from Guizhou (Wangmo) and Yunnan (Cangyuan). However, after examination of the specimens, we found that they were misidentified, and actually belong to *X.distincta* (Fig. 8). According to the information currently available, *M.binigricollis* should be excluded from the fauna of China. *M.binigricollis* needs to be transferred to the genus *Xenicotela* Bates. This issue will be discussed and processed in a separate paper.

### ﻿ Xenicotela convexicollis

Taxon classificationAnimaliaColeopteraCerambycidae

(Gressitt, 1942) comb. nov.

6987D3F9-947D-571C-8C24-274CFE67FF9E

Figs 3 , 4 , 9 , 11 , 16–21

Monochamus convexicollis Gressitt, 1942: 83. Type locality: Zhejiang (Tianmushan), China. Gressitt, 1951: 393; Chou 2004: 296; Hubweber et al. 2010: 282; Lin and Tavakilian 2019: 310.

#### Type material examined.

***Holotype*** (female, IZAS), the label details are shown in Fig. 9.

**Figures 3, 4.**
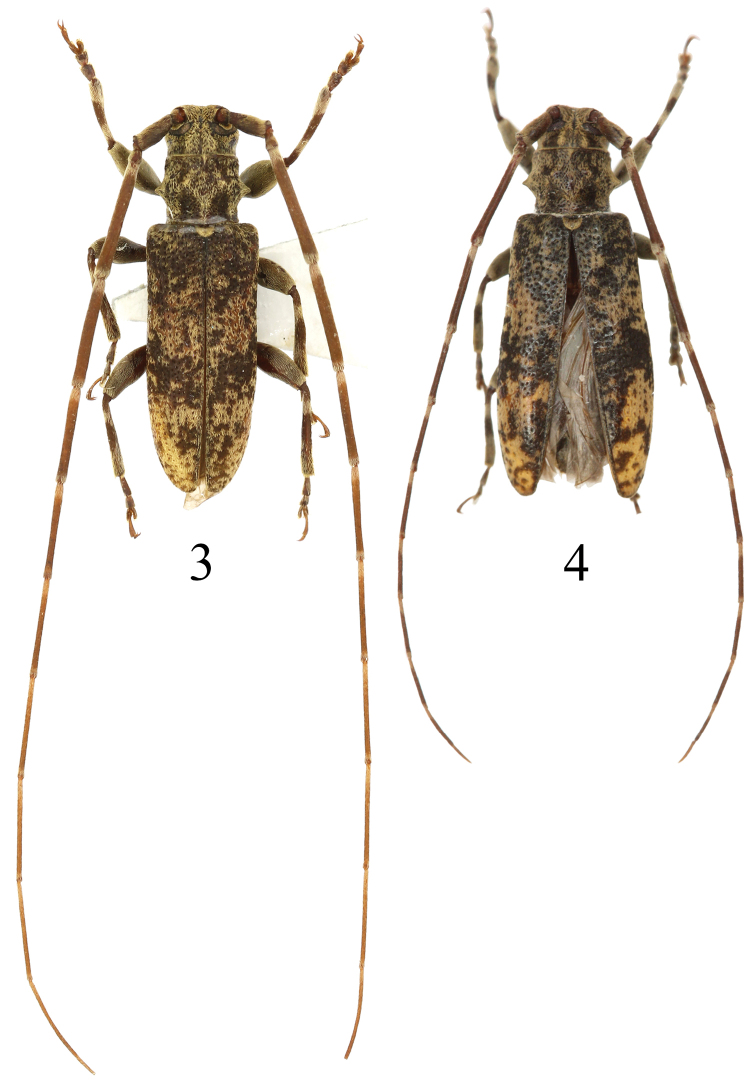
Habitus of *Xenicotelaconvexicollis* (Gressitt, 1942) comb. nov. **3** male **4** female, from Zhejiang: West Tianmushan

#### Other materials examined.

One male and one female: China, Zhejiang, Lin’an, West Tianmushan, July 13, 2012, collected by Guanglin Xie (YZU); one female: China, Zhejiang, Lin’an, Qingliangfeng, May 22, 2012, collected by Guanglin Xie (YZU).

#### Redescription.

**Male.** Body length 11.0 mm, humeral width 3.5 mm. Body mostly black brown, clothed with greyish yellow to pale yellow pubescence, with mottled patches of black and yellow on dorsal surface. Maxillary and labial palpi reddish brown. Antennae dull reddish brown, basal four antennomeres and base of fifth antennomere fringed with very sparse greyish yellow setae, antennomeres III–XI densely annulated with greyish yellow pubescence basally and apically, antennomeres III–V weakly thickened. Pronotum with posterior half furnished with two subparallel longitudinal black stripes of which apex of inner edge bent outward, with anterior half provided with two small stripes obliquely extend outward posteriorly (but indistinct on the holotype). Scutellum clothed with pale yellow pubescence. Elytra unevenly clothed with pale yellow pubescence mottled with various black spots, presenting an incomplete black transverse band behind the middle and mostly black at base. Tibiae black brown, clothed with greyish yellow pubescence forming a subbasal and an apical annulus.

Head finely punctate, frons quadrate, slightly bulging; eyes coarsely faceted, lower lobe longer than broad, about as long as gena. Antennae slender, about 2.8 times as long as body; antennal tubercle moderately raised; scape short, slightly swollen medially; antennomere III distinctly longer than antennomere IV, about 2.5 times as long as scape; antennomeres IV–X nearly equal in length. Pronotum transverse, finely punctate, convex, with centre slightly flat; anterior and posterior margins with vague transverse sulci, each side with a conical spine, short and blunt. Scutellum short, ligulate. Elytra elongated, about 2.3 times as long as width across humeri, with subparallel sides and rounded apices; surface coarsely punctate, the punctures gradually becoming finer towards apex; disc slightly raised at center of basal fourth, followed by a weak central depression along suture. Legs moderately long, with femora slightly swollen medially; prefemur stouter than mesofemur and metafemur; mesotibia without a groove near external apex, metafemur reaching the end of third abdominal segment, claw divaricate.

**Female.** Length 11.0–12.0 mm, humeral width 3.0–3.5 mm. Similar to male, maxillary and labial palpi mostly blackish brown, each side of occiput provided with a black maculation behind upper eye lobe, antennae about 1.8 times as long as body, pronotal lateral spine conical, more cuspidal.

***Male genitalia*.** Tergite VIII (Fig. 11) with both sides converging straight to apex, apex broadly truncated, clothed with short to medium straight setae along apical and lateral sides. Tegmen (Figs 16, 17) length approximately 1.64 mm, maximum width of ringed part approximately 0.52 mm, each paramere length approximately 0.38 mm, basal width approximately 0.17 mm; parameres widely separated at apex, with length-width ratio of each lobe about 2.41, rounded at apex, about apical two-fifths clothed with sparse setae of different lengths and thicknesses. Median lobe (Figs 16, 17) about as long as tegmen, slightly arcuate in lateral view, apical margin of dorsal plate and ventral plate nearly straight; median struts relatively broad, about half length of median lobe.

***Female genitalia*.** Bursa copulatrix (Fig. 21) long, bursiform, slightly expanded apically. Spermatheca (Fig. 21) inserts into the bursa copulatrix at fourth of blind end. Spermathecal duct rather short. Spermathecal capsule approximately S-shaped, tubular, consisting of a basal membranous and an apical strongly sclerotized part, sclerotized tube starts from the second bend and overlaps with membranous part, with blind end slightly curved and expanded. Spermathecal gland located at the joint of membranous and sclerotized part.

#### Distribution.

China (Zhejiang, Taiwan).

#### Comments.

Gressitt (1942) described the species based on a female specimen and originally placed it in the genus *Monochamus* Dejean, 1821. However, after careful examination of the holotype, we conclude that it belongs to the genus *Xenicotela* Bates. This species has the antennae distinctly constricted before the cicatrix, antennomeres III–V clearly fringed with sparse greyish yellow setae ventrally, antennomeres III–XI with the base and extreme apex annulated with greyish yellow pubescence; the pronotum provided with a small, short and conical spine and the mesotibia without a groove, which are well matched with genus *Xenicotela*. Especially, the mesotibia lacks a groove and the antennomeres III–V are clearly fringed with setae, which are obviously different from genus *Monochamus*.

Although the holotype (Fig. 8) does not present black spots on the pronotum, the male and female specimens from the type locality and its adjacent place show distinct black spots on the pronotum (Figs 3, 4). Chou (2004) first recorded this species in Taiwan, China; according to his photographs, there are also distinct black spots on the pronotum. Therefore, we speculate that improper preservation of the holotype may have led to the black spots on the pronotum not being visible.

The species is very similar to *Xenicotelapardalina* (Bates, 1884), however, it can be distinguished from the latter by the lower lobe of the eyes not longer than the gena, and by the elytral base with less light-coloured pubescence, while in *X.pardalina*, the lower lobe is distinctly longer than the gena and the base of elytra is mostly clothed with light-coloured pubescence.

### ﻿ Xenicotela griseomaculata sp. nov.

Taxon classificationAnimaliaColeopteraCerambycidae

138C2DD5-EBC7-53B9-A3FD-5C71EE0AF295

https://zoobank.org/9BB0B30A-2926-4928-91D7-B349FAAC35B5

**Figure 5, 6.**
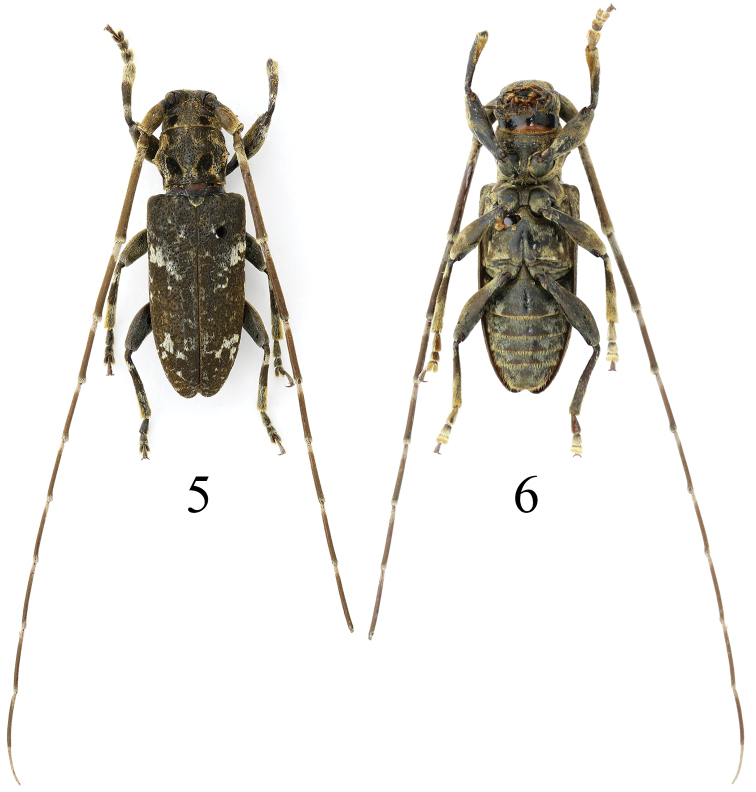
Habitus of *Xenicotelagriseomaculata* sp. nov. Holotype, male, from Chongqing: Wuxi.

灰斑殷天牛 Figs 5 , 6 , 14 , 15 , 24 , 25

#### Type material.

***Holotype***: male, China: Chongqing, Wuxi County, Xiabao township, Shuanghe Village, 31°21'4"N, 109°11'24"E, July 26, 2019, coll. by Bin Chen. The holotype is temporarily stored in the Entomological Museum of Yangtze University (YZU).

#### Description.

**Male.** Body length 12.5 mm, humeral width 4.1 mm. Body mostly black, with greyish yellow, greyish white, brown and black pubescence, with slight mottled maculae. Head with greyish yellow pubescence, denser on gena and around the eyes, and with a subrounded black velvet spot on each side of occiput behind eyes. Antennae mostly clothed with greyish yellow pubescence, fringed with sparse short greyish yellow setae ventrally from first to fifth antennomere; base of scape naked, black, apex of scape and pedicel with slightly greyish white pubescence, bases and extreme apices from antennomeres III–X, base and apical two-fifths of antennomere XI with greyish white pubescence. Pronotum with pubescence greyish yellow mixed with greyish white and brown giving a mottled appearance, each side behind the middle with an oblong black velvet spot edged with mottle of greyish yellow and greyish white, of which the apices obliquely extended outwards and widely separated from each other, the inner edge curved outwards anteriorly. Scutellum with greyish white pubescence, thicker on edge. Elytra with fine and close greyish yellow to brown pubescence, decorated with greyish white pubescent spots as following: each elytron with a conspicuous oblique band after basal fourth, and an incomplete transverse band composed of several irregular spots, before and after the two bands scattered with several small irregular spots. Legs mostly with greyish yellow pubescence, femora only the extreme apex slightly greyish white, tibiae with four pubescent rings alternating black and greyish yellow from base to apex. Ventral surface with non-uniform greyish yellow pubescence, posterior margin of each abdominal sternite fringed with ochraceous pubescence.

**Figures 7–10.**
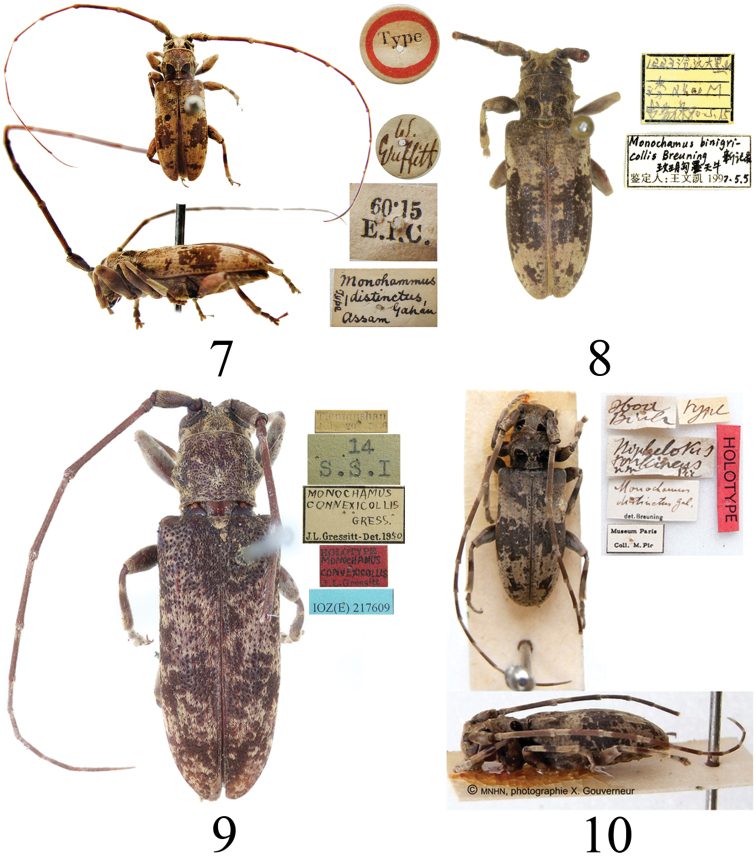
Habitus of *Xenicotela* spp. **7** holotype of *Monohammusdistinctus* Gahan, 1888 **8***Xenicoteladistincta* (Gahan, 1888) **9** holotype of *Monochamusconvexicollis* Gressitt, 1942 **10** holotype of *Nephelotustonkineus* Pic, 1926.

**Figures 11–25.**
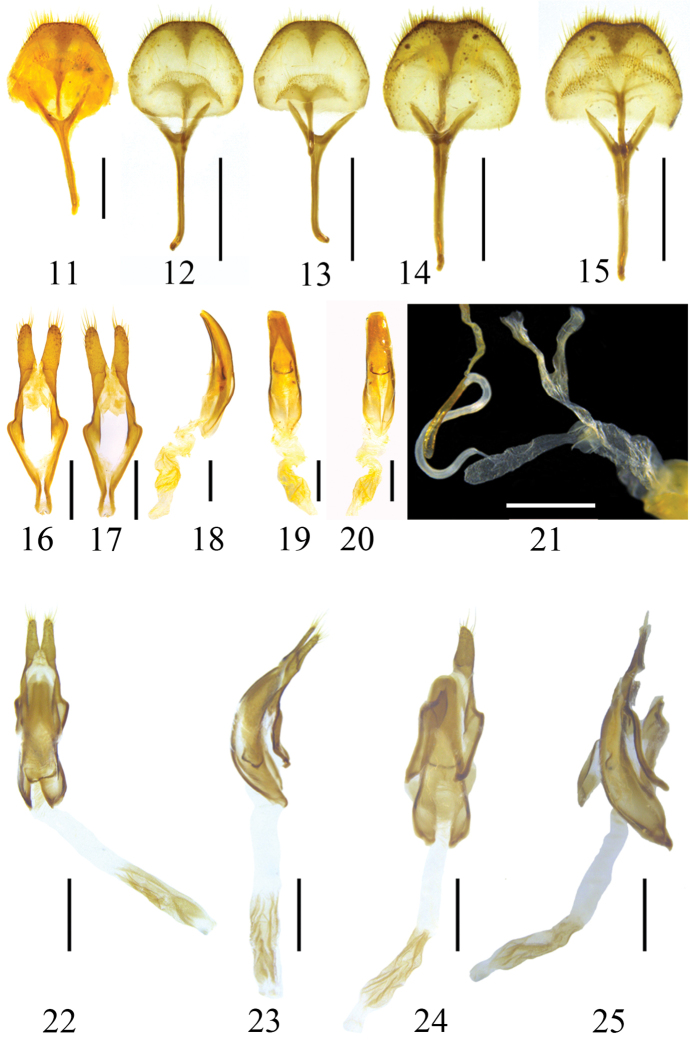
Habitus of *Xenicotela* spp. **11–15** tergite VIII with sternites VIII & IX **16, 17** tegmen **18–20** aedeagus **21** female genitalia **22–25** phallus **11, 13, 15, 17, 19, 24** ventral view **12, 14, 16, 20, 22** dorsal view **18, 23, 25** lateral view **11, 16–21***Xenicotelaconvexicollis* (Gressitt, 1942) comb. nov. **12, 13, 22, 23***Xenicoteladistincta* (Gahan, 1888) **14, 15, 24, 25***Xenicotelagriseomaculata* sp. nov. Scales: 0.5 mm (**11, 16–20**); 1 mm (**12–15, 21–25**).

Head finely and closely punctate, frons transverse, slightly convex, with a smooth fine longitudinal medium sulcus extending to occiput. Eyes coarsely faceted, lower lobe longer than broad, shorter than gena. Antennae slender, about 2.5 times as long as body; antennal tubercles rather elevated, separated from each other; scape stout, slightly flat, with base strongly decrescent and apex distinctly constricted before cicatrix; antennomere III longest, about 2.5 times as long as scape; antennomere IV longer than antennomere V. Pronotum broader than long, anterior margin subequal to posterior margin; each side with a short spine, coniform, blunt apically; disc slightly convex, finely punctate. Scutellum lingulate. Elytra slightly elongated, about 2.0 times as long as width across humeri, gradually narrowing towards apex, apices individually rounded; surface coarsely punctate on base, gradually finer towards apex, middle of basal fourth slightly longitudinally raised. Ventral surface without distinct punctures, procoxal cavities closed posteriorly, mesocoxal cavities open at side, mesosternal process obliquely sloping anteriorly, not tuberculate; apex of terminal abdominal ventrite nearly straight, emarginate medially. Legs moderately long, femora slightly clavate, claws divaricate.

***Male genitalia*.** Tergite VIII (Figs 14, 15) with both sides relatively circularly converge to apex, apex slightly emarginated, clothed with short to medium straight setae along apical and lateral sides. Tegmen (Figs 24, 25) length approximately 1.92 mm, one paramere length approximately 0.48 mm, basal width approximately 0.26 mm, the length-width ratio of paramere about 1.85, rounded at apex, about apical two-fifths clothed with sparse setae of different lengths and thicknesses (tegmen was damaged during dissection and another paramere was lost). Median lobe (Figs 24, 25) slightly longer than tegmen, slightly arcuate in lateral view, apical margin of dorsal plate and ventral plate nearly straight; median struts relatively broad, about two-fifths length of median lobe.

**Female.** Unknown.

#### Distribution.

China: Chongqing.

#### Etymology.

The species is named for the pattern of the elytra, with greyish white pubescent maculae.

#### Comments.

The new species is differentiated from the other species of the genus by the elytra with two incomplete greyish white bands. The new species is similar to *M.binigricollis* in general appearance (it will be transferred to the genus *Xenicotela* in a separate work); however, it can be easy distinguished from the latter by each elytron with apical fourth mostly dull dark brown, furnished with an incomplete greyish white band consisting of several pubescent spots of different sizes, instead of mostly light in colour, dotted with dark spots of various sizes and shapes.

## Supplementary Material

XML Treatment for Xenicotela

XML Treatment for Xenicotela distincta

XML Treatment for Xenicotela convexicollis

XML Treatment for Xenicotela griseomaculata
